# Editorial: 15 years of Frontiers in Cellular Neuroscience: exploring astrocyte heterogeneity: regional functions and impacts on diseases

**DOI:** 10.3389/fncel.2026.1951315

**Published:** 2026-08-11

**Authors:** Carmen Falcone, Francesco Petrelli

**Affiliations:** 1 Biological Sciences, Towson University, Towson, MD, United States; 2 Department of Biomedical Sciences, University of Lausanne, Lausanne, Switzerland; 3 School of Pharmacy, University of Rome “Tor Vergata”, Rome, Italy

**Keywords:** astrocyte, disease, glia, heterogeneity, physiology

Astrocytes were long considered a homogeneous cell population. However, this traditional view has changed dramatically over the past two decades. They differ across species, brain regions, cortical layers, molecular subtypes, and in the reactive states they adopt during disease. This diversity is increasingly recognized as a key determinant of brain function, neural circuit organization, and susceptibility to neurological disorders. As part of the journal's 15-year anniversary series, this Research Topic brings together five contributions that explore the origins, extent, and functional significance of astrocyte heterogeneity, spanning morphology, physiology, computational approaches, and disease.

Two contributions focus on the diversity of astrocyte morphology and its relationship to function in the healthy brain. In their review, Ciani and Falcone examine interlaminar and varicose projection astrocytes, two morphotypes found predominantly in primates and humans. Unlike classical protoplasmic astrocytes, these cells display long, highly organized processes, suggesting that the evolution of astrocyte morphology may have contributed to the emergence of more complex brain functions. Moving beyond morphological description, Freund et al. classified 741 mouse hippocampal CA1 astrocytes into six distinct morphological groups and used detailed multi-compartment biophysical models to investigate their functional properties. They showed that astrocyte morphology strongly influences intracellular calcium responses to glutamate, affecting the amplitude and kinetics of calcium signals, whereas sodium and potassium dynamics were largely preserved across morphotypes. Together, these findings provide a direct mechanistic link between astrocyte structural heterogeneity and functional diversity. Two additional studies highlight how astrocyte heterogeneity emerges in disease. Using a mouse model of Huntington's disease, Brown et al. showed that GFAP^+^ and S100B^+^ astrocytes represent largely distinct populations that respond differently to pathology. In particular, GFAP^+^ astrocytes selectively accumulated in the dorsomedial striatum, where they clustered around white

matter fascicles and were enriched in regions with a relatively low burden of mutant huntingtin aggregates. These findings emphasize the importance of considering both astrocyte subtype and anatomical location when studying reactive astrogliosis. Focusing on ischemic brain injury, Riew et al. demonstrated that reactive astrocytes, rather than microglia, are the main source of osteopontin in the hippocampus. Together with S100β, astrocyte-derived osteopontin promotes the formation of corpora amylacea-like structures from degenerating neuronal debris, supporting an active role for reactive astrocytes in tissue remodeling and debris clearance after injury.

Finally, Pérez et al. extend the concept of astrocyte heterogeneity to the subcellular level. Using graph-theoretical analysis of astrocytic calcium activity, they propose that individual astrocytic processes function as specialized input, output, and hub compartments, generating a hierarchical and directional flow of information. Their Perspective suggests that this functional organization is dynamically remodeled during development, aging, and disease, highlighting astrocytes as compartmentalized computational units rather than passive integrators. Together, these studies illustrate that astrocyte heterogeneity is a fundamental feature of brain organization, spanning multiple biological scales, from differences between species, brain regions, and molecular subtypes to functional specialization within individual cells. They also demonstrate how advances in imaging, molecular profiling, morphological reconstruction, and computational modeling are providing new opportunities to investigate astrocyte diversity and function. Understanding how astrocyte heterogeneity is established, maintained, and altered during disease will be essential for defining its biological significance and for identifying new therapeutic opportunities for neurological disorders. More broadly, these contributions reinforce the view that astrocyte heterogeneity is not merely a descriptive feature, but a fundamental principle of astrocyte biology that shapes brain function in both health and disease. We sincerely thank all the authors, reviewers, and readers who contributed to this Research Topic.

